# Supplementary material for: Content-rich biological network constructed by mining PubMed abstracts
Source: BMC Bioinformatics. 2004 Oct 8;5:147. doi: 10.1186/1471-2105-5-147 (PMC528731; doi:10.1186/1471-2105-5-147)
Supplement: Additional File 2 — The original results of the above study (non-essential files are deleted to keep the file size under the limit set by BMC bioinformatics). [file 1471-2105-5-147-S2.bz2 › chilibotAdditionalFile2/dip10/713ID8175697E18050/html/SECA_SECB.html]

 


 **SECA** and **SECB** 
  
Found 76 abstracts in PubMed, retrieved 10.  
 

 What does Google say? 
 PDF only 
| .edu only 

---

**Interactive relationship** (e.g. stimulation, inhibition, etc)

**Stimulatory relationship**- The equilibrium dialysis experiments and kinetic data support the observation that the  **SecB**  enhanced  **SecA**  ATPase activity is the result of an increased rate of ATP hydrolysis rather than an increase in the affinity of ATP for  **SecA** .  Ref: 11955083 Biochemistry, 2002
**Neutral relationship**- In Escherichia coli, the formation of  **SecA**   **SecB**  complexes has a direct effect on  **SecA**  ATPase activity.  Ref: 11955083 Biochemistry, 2002
- In this latter role  **SecB**  demonstrates specific binding to a unique partner,  **SecA** .  Ref: 12475171 Cell Mol Life Sci, 2002
- It appears that  **SecB**  may function as an intermolecular regulator of ATP hydrolysis by promoting the ATP bound state of  **SecA** .  Ref: 11955083 Biochemistry, 2002

**Non-interactive relationship** (e.g. studied together, co-existance, homology, etc.)

- The incorporation of sequence motifs in the mature region, which confer  **SecB**  dependence in vivo, had no impact on  **SecA**  activation in vitro.  Ref: 11969418 Biochemistry, 2002
- The inhibition of  **SecA**  ATPase activity by sodium azide in the presence of IMVs and a functional signal peptide further indicates that  **SecB**  promotes the ATP bound form of  **SecA** .  Ref: 11955083 Biochemistry, 2002
- In Escherichia coli, the  **SecB**   **SecA**  branch of the Sec pathway and the twin arginine translocation Tat pathway represent two alternative possibilities for posttranslational translocation of proteins across the cytoplasmic membrane.  Ref: 12700260 J Bacteriol, 2003
- In Escherichia coli, three different types of proteins engage the SecY translocon of the inner bacterial membrane for translocation or insertion 1 polytopic membrane proteins which prior to their insertion into the membrane are targeted to the translocon using the bacterial signal recognition particle SRP and its receptor, 2 secretory proteins which are targeted to and translocated across the SecY translocon in a  **SecA**  and  **SecB**  dependent reaction and 3 membrane proteins with large periplasmic domains, requiring SRP for targeting and  **SecA**  for the translocation of the periplasmic moiety.  Ref: 12682042 J Biol Chem< MedlineTA>J Biol Chem, 2003
- Export after  **SecA**  depletion was observed in cells that lack  **SecB** , the primary targeting factor for OMPs, but was abolished by partial inactivation of DnaK.  Ref: 12403776 J Biol Chem, 2002
- The characterization of a spontaneous TorA signal peptide mutant TorA\*, in which the two arginine residues in the c region had been replaced by one leucine residue, showed that the TorA\* MalE mutant precursor had acquired the ability for efficiently using the  **SecB**   **SecA**  pathway.  Ref: 12700260 J Bacteriol, 2003
- **SecB**  modulates the nucleotide bound state of  **SecA**  and stimulates ATPase activity.  Ref: 11955083 Biochemistry, 2002
- The proteolysis data indicate that  **SecB**  and the nonhydrolyzable ATP analogue AMP P C P induce similar conformational changes in  **SecA**  which result in a more open or extended structure that is suggestive of the ATP bound form.  Ref: 11955083 Biochemistry, 2002
